# Human values, civic participation, and wellbeing: analysis on their relationship among older Europeans

**DOI:** 10.3389/fpsyg.2024.1346730

**Published:** 2024-03-07

**Authors:** Andrea Vega-Tinoco, Julia Sánchez-García, Marta Gil-Lacruz, María José Sierra Berdejo, Ana Isabel Gil-Lacruz

**Affiliations:** ^1^Department of Business Direction and Organization, University of Zaragoza, Zaragoza, Spain; ^2^Department of Psychology and Sociology, University of Zaragoza, Zaragoza, Spain

**Keywords:** Schwartz Basic Values, civic engagement, older adults, well-being, pseudo-panel, Europe

## Abstract

**Introduction:**

So far, both for the general and older population, research on human values and wellbeing mainly shows correlational associations but does not inquire about the direction of this relationship. This is also true for values and civic participation. Therefore, our objective is to identify the directional association between civic participation and Schwartz values, and between values and wellbeing, among older Europeans.

**Methods:**

A pseudo-panel was created from the cross-sectional data of the European Social Survey (ESS 2002-2018), controlling for gender, age-group, country and level of studies (*n* = 3926). The data analysis was performed using a cross-lagged model, applying both random-effects and fixed-effects models.

**Results:**

On the one hand, the relationship between participation and values is bidirectional, but the effect of civic participation on values is more significant since participating stimulates the development of certain values. On the other hand, although the relationship between values and wellbeing is also bidirectional, the effect of wellbeing on values is stronger since a given level of wellbeing favors the development of particular values.

**Discussion:**

We conclude that civic participation should be promoted within the older population since it directly increases wellbeing, and moreover reinforces those (Growth-oriented) values that positively influence the health, happiness and life satisfaction of older people.

## 1 Introduction

The extension of life expectancy and reduced fertility rates in economically developed societies have resulted in major demographic changes that have led to population aging worldwide. Even middle- and low-income countries are now joining this trend (World Health Organization, 2021). The case of Europe stands out, with an estimated quarter of its population expected to be over 65 years of age by 2050 (United Nations, 2019). European countries lead the world in terms of the share of older people among their population (Balachandran et al., 2020).

Happily, these data reflect an achievement in reaching the fundamental goals of a society (Götmark et al., 2018) and thus an improvement in its quality of life. Moreover, older adults today have fewer disabilities and are healthier than their earlier counterparts (Balachandran et al., 2020). Nevertheless, population aging poses a significant challenge to health, retirement, and social protection systems (Gusmano and Okma, 2018; United Nations, 2019). Therefore, it requires new and innovative solutions that lead this growing collective to achieve high levels of wellbeing through active aging, which in turn have an impact on greater wellbeing for the societies in which they are integrated.

Likewise, the current extension of physical and mental wellness in old age facilitates the civic participation of people aged 65 and over. As already evidenced by the multiple benefits of volunteering (see Haski-Leventhal, 2009; Wray-Lake et al., 2017; Proulx et al., 2018), civic participation is also a potential resource for promoting the wellbeing of older people (Wray-Lake et al., 2017; Serrat et al., 2020; Vega-Tinoco et al., 2021, 2022). It is therefore important to understand what motivates such participation.

Values are motivational constructs that represent broad goals that endure across diverse contexts and moments (Sagiv and Schwartz, 2022). As such, human values could be one of the factors that drive older adults to engage in civic activities. Schwartz (2010) expresses that the importance assigned by a person to certain values can promote or inhibit their pro-social behavior. However, it is also worth wondering whether a person with an orientation towards certain values is more likely to be involved in civic activities, or reverse-wise, is it such involvement that fosters an inclination towards certain values?

Thus, the central objective of the present research is to provide evidence to better understand the relationship between the importance that older people assign to different human values and their propensity to civic participation. So far, most of the research related to values and civic engagement is based on correlational and cross-sectional studies (Veeh et al., 2019). Therefore, the main contribution of this paper is to give the research a longitudinal nature that can shed some light on the direction of the relationship between human values and civic engagement. Likewise, since civic participation is linked to wellbeing as its ultimate goal, this study also aims to identify the directional association between human values and wellbeing.

The Schwartz Theory of Basic Human Values has been taken as the model for the present work due to its widely proven validity within the scientific literature (Reeskens and Vandecasteele, 2017; see Grosz et al., 2021; Hamby and Farrell, 2022; Sagiv and Schwartz, 2022; Schwartz and Cieciuch, 2022) and for being the most applied approach in social sciences (Sagiv and Schwartz, 2022). This model has also served as the basis for the development of the European Social Survey (ESS) which, again, is a widely used and academically validated cross-national survey that collects information about the beliefs, attitudes and behaviors of more than thirty populations around the world.

The structure of this paper consists of a first section in which a review of the scientific literature on civic participation, human values and the wellbeing of the elderly is carried out, from which the starting hypotheses are derived. This is followed by a description of the materials and methods, the database, the main variables and the analytical strategy. Results are then introduced, first in their descriptive form, and afterwards with regard to the regression analysis. The resulting relationships between variables—as well as their implications—are then discussed and contrasted with the findings of other authors, followed by the validation or rejection of the hypotheses. Finally, the limitations of this study and a proposal for future lines of research are presented.

## 2 Literature review

### 2.1 Civic participation and active aging

In view of the demographic, economic and social changes brought about by population aging, the notion of active aging implies salutogenic strategies to be implemented throughout the life cycle with the aim of extending a person’s healthy life expectancy and quality of life (World Health Organization, 2002). In Europe, this premise has guided the scientific conceptualization of wellbeing during old age and has channeled the design of concordant public policies (Foster and Walker, 2015).

The wellbeing of older adults goes beyond the claim of reaching old age in good physical condition. Wellbeing encompasses health in a broader sense, including the physical, mental and social dimensions (World Health Organization, 2002). Among the various indicators that denote well-being, a person’s perception of their own quality of life (which translates into subjective wellbeing), self-assessed in both the short term (happiness) and long term (life satisfaction), is rather relevant (Helliwell and Putnam, 2004; VanderWeele et al., 2020). Consequently, in this research, health, happiness, and life satisfaction are understood as indicators of the wellbeing of the older population.

Likewise, the concept of active aging also transcends the need to prolong the involvement of older people in the labor market (Foster and Walker, 2015). A holistic approach encompasses the participation of the person in society, in their families and in their communities, through activities that are in line with their desires, capabilities, needs and opportunities (World Health Organization, 2002; Rantanen et al., 2019). Similarly, Helliwell et al. (2019) assert that a person (especially those over 60 years of age) feels greater satisfaction with life to the extent that they feel involved in their community. Furthermore, participating within the community is associated with greater mental wellbeing (Ding et al., 2015) and with high social trust, belongingness, and community bonding (Veeh et al., 2019). Thus, one might conjecture that civic engagement may also represent an opportunity for active aging. Nonetheless it is not only the older adult who benefits from such participation, but also the society, in terms of gains in experience and generativity, supportive roles for families and communities, and the creation and preservation of social capital (Villar and Serrat, 2014; Villar et al., 2021).

Civic participation refers to activities, political or social, carried out by ordinary citizens that aim to improve society, care for others, or directly or indirectly influence the actions of those in power (Verba et al., 1978; Thomassen, 2003; Ekman and Amnå, 2012). In this work, and in line with the European Social Survey, the following undertakings are considered as participatory activities: signing petitions, wearing campaign badges, contacting politicians, boycotting products, demonstrating publicly, or voluntary participation in political and non-political organizations. Regarding the different ways of civic participation, there is extensive literature that endorses the benefits obtained by older people who get involved in volunteering. Examples include improved life satisfaction, perceived health and life expectancy, in addition to reducing depression (Haski-Leventhal, 2009), preventing cognitive decline (Proulx et al., 2018) and promoting the wellbeing and development of their communities (Gonzales et al., 2015).

Despite the scarce scientific literature showing the benefits of civic participation on the wellbeing of the older population, previous studies have shown that such participation has positive repercussions on their happiness, health and life satisfaction (Vega-Tinoco et al., 2021), in addition to its key role as a means for older people to stay active and socially involved, and to have their opinions taken into account and represented in the political spheres (Serrat et al., 2020). Moreover, the literature is scarce regarding the motivations of older adults to participate in this type of civic activities. Hence, this paper seeks to analyze the fundamental values proposed by Schwartz as possible motivators of such participation.

### 2.2 The Schwartz Theory of Basic Human Values

Schwartz (1992) defines human values as desirable and transcendent goals that serve as principles that guide people’s lives, according to the individual importance assigned to each, and that motivate behaviors, perceptions, and attitudes (Schwartz, 2003; Schwartz and Cieciuch, 2022). The type of motivational goal that a value expresses is its central core, a key aspect that differentiates the fundamental values proposed by the author. Even among different cultures, it can be found that virtually all specific values can fit within the same integrative model. The 10 universal values proposed by Schwartz (1992) are: Benevolence, Universalism, Self-direction, Stimulation, Hedonism, Achievement, Power, Security, Conformity, and Tradition. Table 1 shows these core values along with a description of their primary motivational goal.

**Table 1:** Schwartz ten basic human values and their motivational goals.

Value	Motivational goals
*Achievement*	Personal success, demonstration of capabilities that meet social standards
*Power*	Social status, prestige, and control over people and resources
*Security*	Stability, security and harmony of society, relationships and oneself
*Conformity*	Restraint of actions and impulses that may harm or upset others, or violate social expectations or rules
*Tradition*	Respect, commitment and acceptance of the customs and ideas dictated by traditional culture or religion
*Benevolence*	Concern for and enhancement of the wellbeing of people with whom one is in frequent contact
*Universalism*	Concern, understanding and protection of the wellbeing of all people and of nature
*Self-Direction*	Independence of thought and action, and also to choose, create, and explore
*Stimulation*	Novelty, excitement and challenges throughout life
*Hedonism*	Pleasure and gratification of one’s senses

Adapted from Values and subjective well-being (pp. 3–4), by S. H. Schwartz & F. M. Sortheix, 2018, in E. Diener, S. Oishi, & L. Tay (Eds.), Handbook of well-being. DEF Publishers. DOI: nobascholar.com.

**Figure 1:**
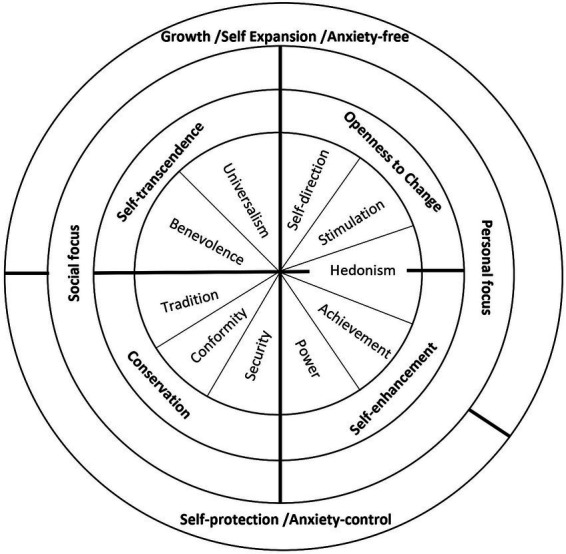
The structural model of the Schwartz theory of basic values: circular structure of the ten basic human values, four higher-order dimensions, and two underlying motivational sources. Note: Adapted from Values and subjective well-being (p. 3), by S. H. Schwartz & F. M. Sortheix, 2018, in E. Diener, S. Oishi, & L. Tay (Eds.), Handbook of well-being. Salt Lake City, UT: DEF Publishers. DOI: nobascholar.com.

The Theory of Human Values describes the dynamic relationships that occur between these values, i.e., it posits a structural model. As can be seen in Figure 1, the circular arrangement of the values expresses the motivational continuity of the structure: the greater the similarity of the core motivations of the values, the closer they will be within the circle (in either direction) and can be pursued by the same action. Likewise, the greater the opposition of these motivations, the more distant the values will be and, in general, they cannot be pursued by the same action. Those values located at opposite ends of the circle then contain opposing central motivations (Schwartz, 1992, 2003, 2012; Schwartz and Cieciuch, 2022).

Likewise, such a structure is divided into four higher-order categories. Openness to Change emphasizes autonomy and independence, as well as willingness for new experiences. Conservation refers to the maintenance of the established order, self-control and resistance to change. Self-enhancement comprises the pursuit of prestige, success and self-interest, while Self-transcendence is concerned with the interests and wellbeing of others (Schwartz, 2003; Egri and Ralston, 2004). Figure 1 shows the values that belong to each category, as well as the organization and relationship of the structure.

Additionally, this theory contemplates the principle of the interest that the values serve, since they can be oriented towards a social focus or a personal focus. The values of Universalism, Benevolence, Tradition, Conformity and Security (left side of the figure) regulate the way in which a person relates socially and to the interests of others. Whereas Self-direction, Stimulation, Hedonism, Achievement and Power (right part of the figure) regulate the way in which one expresses one’s own interests and personal characteristics. It should be emphasized that Security and Universalism are borderline values between the social and personal approaches, as their orientation is primarily directed towards others, but they also regulate the pursuit of self-interest. The last principle is based on the relationship of values to the anxiety caused by uncertainty in the social and physical world. Self-protection values attempt to cope with this anxiety, while Growth values express anxiety-free motivations (Cieciuch et al., 2015; Schwartz and Cieciuch, 2022).

In his later work, Schwartz (2017) refines the theory and proposes 19 different values, but the author himself acknowledges that his model presents a non-binding character due to the continuous nature of motivations. The author emphasizes that it is advisable to divide the value items into a greater or lesser level of fineness, corresponding to the needs and objectives of every study (Schwartz, 2017). Thus, for the present analysis we consider more convenient the 10-value model so that similarity between a value and its contiguous ones is avoided to a greater extent.

### 2.3 Values, civic participation and wellbeing

The consideration of values as a structure increases the possibility of predicting and understanding the relationship they have with other variables such as, for example, people’s behavior, opinions, attitudes or social experiences. If a value has a significant impact on a variable, it is very likely that its adjacent and opposite values also have a relevant association with that variable (Schwartz, 2003, 2012).

Schwartz (2010) indicates that there is a positive association between volunteering and pro-social behavior, characterized by the values of Universalism, Benevolence and Conformity. Likewise, there are several studies that find a positive correlation between volunteering, social awareness and other pro-social actions with the values of Self-transcendence (Universalism and Benevolence) and, on the contrary, a negative correlation with Power and Achievement (Pepper et al., 2009; Plagnol and Huppert, 2010; Bathini and Vohra, 2013). Schwartz (2010) also indicates that values denoting individualism (Power and Security) seem to hinder pro-social behavior. However, if a person engages in volunteering activities for the sake of gaining public recognition or social acceptance, Self-enhancement values might not only not hinder volunteering, but promote it (Ariza-Montes et al., 2017). As volunteering is one of several forms of civic participation, it seems reasonable to think that values might have similar correlations with this participation as well.

The study by Funk (1998) posed a similar question: does people’s value orientation help explain their level of civic involvement in their communities? The results revealed that individuals whose values are oriented towards others (Self-transcendence) are more likely to participate civically. Accordingly, people who showed greater individualism reported a lower propensity to engage in such activities. Bekkers (2005) also reports that civically active citizens manifest values with more post-materialistic orientations.

The preliminary results of Hamby and Farrell (2022) also reflect that values related to achievement and growth may reduce the intention to engage in civic activities. Moreover, they indicate that the reasons that lead a person to engage in pro-social experiences are often based on their underlying values, which in turn may influence the person’s response to the participatory experience and their satisfaction with it. The authors also point out that differences in value orientation, as a motivation for engaging in such activities, may explain why this type of activity does not affect everyone equally. However, there is still insufficient scientific literature studying the relationship between human values and civic participation. And, above all, the existing studies show the association between values and participation (correlation), but do not inquire about the direction of this relationship. This is also true for the relationship between values and wellbeing.

In addition to the empirical evidence that supports the link between civic participation and wellbeing, the direct relationship between wellbeing and fundamental values is still pending. In this field, the documentation on the values-wellbeing link is ample, but its directional association has not yet been established.

The scientific literature states that both directions of the wellbeing-values relationship are possible. On the one hand, as proposed by Sortheix and Schwartz (2017) by unifying the theories existing until then, it is possible that pursuing values that have been considered as “healthy” and that also satisfy intrinsic and self-growth and actualization needs (e.g., Openness to Change) lead to higher subjective wellbeing. Accordingly, values that are considered “unhealthy” and that require stressful activities for self-protection (e.g., Conservation) have the opposite effect. The study by Sagiv and Schwartz (2000) found similar associations and further notes that values seem to have a rather weak but direct influence on wellbeing.

On the other hand, it is also possible that the association goes in the other direction, so that the level of subjective wellbeing influences the importance assigned to certain values. It is possible that a person with a high level of wellbeing has the resources to pursue the autonomy and concern for others represented by the values of Self-direction, Benevolence, Stimulation and Universalism. Whereas a person who does not enjoy such wellbeing may be more focused on their own problems as to pursue these types of values, giving more priority to others such as Security and Power (Sagiv and Schwartz, 2000; Schwartz and Sortheix, 2018).

However, although there is a large number of cross-sectional studies on the association between values and subjective wellbeing, there is barely any longitudinal research on their temporal interaction (Grosz et al., 2021). Therefore, this paper also pays special attention to the possible direction of the relationship between values and wellbeing among older people.

Based on the above, the hypotheses of this study are as follows:

*H1:* The relationship between civic participation and human values is bidirectional, with the impact of values on participation being stronger than vice versa.

*H2:* The relationship between wellbeing and human values is bidirectional, with the impact of values on wellbeing being stronger than vice versa.

*H3:* Self-transcendence values promote civic participation, while Self-enhancement values hinder it.

*H4:* Openness to Change values promote wellbeing, Conservation hinders it, and Self-transcendence and Self-enhancement present a mixed association (both positive and negative).

## 3 Materials and methods

### 3.1 Data

The European Social Survey (ESS) is a pan-European research infrastructure that provides freely accessible data for academics, policymakers, civil society, and the wider public. The organizational structure of ESS is characterized by its cross-national nature and the coordination of efforts from academics at various institutions including the University of London (leading role), the Leibniz Institute for the Social Sciences, the Norwegian Agency for Shared Services in Education and Research, the Netherlands Institute for Social Research, Universitat Pompeu Fabra, the University of Essex, and the University of Ljubljana. The ESS is a comprehensive research tool that provides accessible data for academics, policymakers, civil society and the wider public. It is designed to observe shifts in European attitudes and values over time (ESS, 2023).

The ESS adheres to rigorous sampling methods to ensure representative data across all participating nations. A strict no-substitution policy for non-respondents is maintained to preserve data integrity. Each country aims for a minimum effective sample size of 1,500 with response rates targeted at 70%, although there are variations among countries across waves. Finland, Germany, Hungary, Norway, and Sweden have experienced a decline in response rates, although it is never consistent and never below 60%. On the other hand, France, Spain, and Switzerland have shown a remarkable increase in response rates to 70%. Response rates in Belgium, Poland, and the United Kingdom remain stable at around 70%. In Ireland, The Netherlands, and Portugal, however, response rates can be quite erratic, ranging from 60 to 70%. The ESS is committed to producing reliable and comparable data across Europe, which facilitates in-depth analysis of societal trends. This meticulous approach ensures the accuracy of the data (Beullens et al., 2018).

The cross-sectional data were taken from the 9 waves of the European Social Survey, collected every 2 years from 2002 to 2018 (ESS, 2002–2018). Only persons born before 1965, resident in Belgium, Finland, France, Germany, Hungary, Ireland, Netherlands, Norway, Poland, Portugal, Spain, Sweden, Switzerland and the United Kingdom were considered.

### 3.2 Variables

As the main objective of this study is to find evidence on the direction of the relationships, all variables have been considered as dependent or independent according to need. The three main groups of variables are: basic values, civic participation and wellbeing.

An individual variable was assigned for each of the 10 values (*Benevolence, Universalism, Self-direction, Stimulation, Hedonism, Achievement, Power, Security, Conformity* and *Tradition*), as well as a variable for each of the 4 dimensions (Conservation, Self-transcendence, Openness to Change and Self-enhancement). The ESS consists of between 2 and 3 indexes for each value, to which the respondent answers on a scale from 1 to 6 if that value is “not at all like me” to “very much like me.” The score for each variable value is equal to the mean of the indexes corresponding to that value. Also, for each dimension we take the mean of the values that conform it.

In addition, the ESS asks whether the person has done any of the following activities in the last 12 months in order to “improve things in [their country] or help prevent things from going wrong”: signing petitions, wearing campaign badges or stickers, contacting politicians or government officials, boycotting products, demonstrating, or working in a political party or other type of organization/association. *Civic participation* results in a single dichotomous variable, which takes the value of 1 if the person has been involved in any activity, and 0 if not.

Also, *health*, *happiness* and *life satisfaction* are taken as indicators of the wellbeing of older people. The corresponding questions in the ESS are: “Taking all things together, how happy would you say you are?” “All things considered, how satisfied are you with your life as a whole nowadays?” and “How is your health in general?” These are answered on a scale from “Extremely unhappy/unsatisfied” (0) to ““Extremely happy/satisfied” (10); as well as from 1 to 5, meaning “Very bad” to “Very good” health (the scale has been inverted to simplify the interpretation of the results, being the original ESS scale from 1 = “Very good” to 5 = “Very bad”). Helliwell and Putnam (2004) confirm that self-reported variables are a valid and reliable measure of subjective wellbeing. In the end, the three variables (*health*, *happiness* and *life satisfaction*) have been reconstructed in a dichotomous way (1 = high wellbeing, 0 = otherwise), considering that a person’s level of wellbeing is high if the interviewee scores 9 or 10 for happiness and satisfaction, and 4 or 5 for health.

### 3.3 Analytical strategy

In the absence of panel data at the individual level, a pseudo-panel gives the research a longitudinal character and enables the study of the changes over time of the variables of interest (Deaton, 1985). Thus, we have grouped people with the same time-invariant characteristics: gender (male or female), age-group, country and level of studies. We accounted for 6 age-groups: early Silent Generation (born between 1936 and 1940); mid Silent Generation (1941–1945); late Silent Generation (1946–1950); early Baby Boomers (1951–1955); mid Baby Boomers (1956–1960); and late Baby Boomers (1961–1965). Also, level of studies (1 = primary or less, 2 = secondary, 3 = tertiary) is considered time-invariant given the age range of the individuals.

The pseudo-panel design comprises the construction of cohorts that group together individuals who meet the same characteristics, representing a “standardized person.” For example, one cohort could represent a “female, late Silent Generation, tertiary level of education in Sweden.” Thus, each variable will take the mean value of the individual responses of the people who form that cohort, and hence the changes of that “standardized person” can be tracked over time (Deaton, 1985; Guillerm, 2017; Reeskens and Vandecasteele, 2017). After forming cohorts (with no less than 30 individuals per cohort), our final sample consists of 3,926 observations, i.e., 437 cohorts (with x̄=235 individuals per group; σ = 163) across the 9 waves (2002–2018).

In order to infer directional associations with longitudinal data, we used a cross-lagged model (Imai and Kim, 2019; Russell et al., 2019), applying both random-effects (RE) and fixed-effects (FE) models. We ran the FE model suggested by the Hausman test, however, this model can be highly dependent on sample size and therefore we opted to run the RE as well, so as to minimize variance. Although the RE coefficients may be overestimated, they also provide more significant results and the use of both models will also shed more light on their interpretation. The software used was STATA 14 [Stata (RRID:SCR_012763)], commands *xtreg, re* and *xtreg, fe*.

## 4 Results

The results of the descriptive statistics and regressions are presented below. Only statistically significant results are described.

### 4.1 Descriptive statistics

Descriptive statistics show that the values to which older adults assign the greatest importance are *Benevolence* (x̄=4.80/6) and *Universalism* (x̄=4.71/6), while, on the contrary, the values to which they attribute the least importance are *Power* (x̄=2.99/6), *Stimulation* (x̄=3.23/6) and *Achievement* (x̄=3.39/6). In addition, 60% of older European consider themselves to be in good or very good health, and 28% report being happy and 26% are satisfied with their lives (Table 2).

**Table 2:** Descriptive statistics for civic participation, 10 basic human values and wellbeing indicators.

Variable	Mean^a^	Std. Dev.	Min	Max
*Civic participation*	0.52	0.24	0.00	1.00
*Tradition*	4.18	0.53	0.00	6.00
*Conformity*	3.95	0.53	0.00	6.00
*Benevolence*	4.80	0.49	0.00	6.00
*Universalism*	4.71	0.48	0.00	5.92
*Security*	4.41	0.60	0.00	6.00
*Self-direction*	4.47	0.50	0.00	6.00
*Power*	2.99	0.48	0.00	5.00
*Achievement*	3.39	0.57	0.00	5.50
*Hedonism*	3.71	0.60	0.00	5.50
*Stimulation*	3.23	0.47	0.00	5.50
*Health*	0.60	0.21	0.00	1.00
*Happiness*	0.28	0.15	0.00	1.00
*Life satisfaction*	0.26	0.16	0.00	1.00

Total observations = 3,926 individuals, grouped in 437 cohorts.

a Civic participation scores (scale from 0 to 1) are equivalent to the percentage (%) of people who wave participated in the last year. Wellbeing scores (scale from 0 to 1) are equivalent to the percentage (%) of people with high levels of wellbeing. Human values scores range from 0 (value not at all important) to 6 (value extremely important).

Also, 52% of the participants reported having carried out some kind of civic activity in the last year. However, the sample can be divided according to the 4 higher-order dimensions of the Schwartz theory, assigning to each person a dimension marked by the values they consider most important. Thus, we see that the older people who participate the most are those who tend towards *Self-enhancement* and *Openness to Change*, with 55 and 54% of these people having participated, correspondingly. Likewise, the highest percentage of older adults who express having good health are those who lean towards *Self-enhancement*, and the most satisfied are those who lean towards *Openness to Change*. On the other hand, the people who tend towards *Conservation* report lower health, and *Self-transcendence* report lower life satisfaction. The proportion of happy individuals does not seem to vary meaningfully by dimension (Table 3).

**Table 3:** Mean scores of civic participation and wellbeing by human values dimension.

Variable	Conservation		Self-enhancement		Self-transcendence		Openness to change	
	Mean	Std. Dev.	Mean	Std. Dev.	Mean	Std. Dev.	Mean	Std. Dev.
*Civic participation* ^a^	0.44	0.24	0.55	0.24	0.44	0.24	0.54	0.23
*Health* ^b^	0.56	0.22	0.63	0.21	0.59	0.22	0.61	0.21
*Happiness* ^b^	0.26	0.15	0.27	0.14	0.24	0.16	0.28	0.15
*Life satisfaction* ^b^	0.24	0.16	0.26	0.17	0.22	0.15	0.27	0.17
**Total observations**	753		308		172		2,693	

a Mean scores represent the percentage of people who have participated in the last year.

b Mean scores represent the percentage of people with high levels of wellbeing.

Furthermore, civic engagement, wellbeing and human values scores vary as people age. 53% of middle-aged persons have been involved in *civic participation*, but this percentage declines with age, dropping to 41% by the time they reach the fourth-age. *Health* also deteriorates with age, but the difference in *happiness* and *life satisfaction* is not really substantial between the middle- and fourth-age, although a positive peak does occur at the third age. In terms of human values, people seem to give more importance to *Conservation* values as they age, and less to *Self-enhancement* and *Openness to Change* (Table 4).

**Table 4:** Civic participation, wellbeing and human values dimension by age group.

Variable	Middle-aged		Third-age		Fourth-age	
	(50–64 years old)		(65–79 years old)		(80+ years old)	
	Mean^a^	Std. Dev.	Mean	Std. Dev.	Mean	Std. Dev.
*Civic participation*	0.53	0.23	0.50	0.25	0.41	0.28
*Health*	0.64	0.19	0.53	0.22	0.40	0.26
*Happiness*	0.27	0.14	0.30	0.17	0.26	0.19
*Life satisfaction*	0.24	0.15	0.30	0.19	0.27	0.21
*Conservation* ^b^	0.16		0.21		0.25	
*Self-enhancement* ^b^	0.08		0.6		0.03	
*Self-trascendance* ^b^	0.02		0.2		0.02	
*Opennes to change* ^b^	0.74		0.71		0.70	
**Total observations**	2,541		1,321		64	

a Civic participation scores (scale from 0 to 1) are equivalent to the percentage (%) of people who wave participated in the last year. Wellbeing scores (scale from 0 to 1) are equivalent to the percentage (%) of people with high levels of wellbeing.

b Dimension scores (scale from 0 to 1) are equivalent to the percentage (%) of people who find said dimension to be the most important. All 4 Dimension scores sum to 1.

Regarding the values-participation correlation, *Tradition*, *Conformity*, *Security*, *Power* and *Achievement* have a negative correlation with *civic participation*. In contrast, the relationship is positive with *Benevolence*, *Universalism*, *Self-direction*, *Hedonism* and *Stimulation*. Also, the strongest correlations between values correspond to those that are close within the continuous circle proposed by Schwartz, and the opposite values show weaker correlations, confirming the theoretical structure. For example: *Self-direction* has the strongest correlation with *Universalism* (0.73), then *Benevolence* (0.69) and *Stimulation* (0.67), while it correlates the weakest with *Conformity* (0.30) and *Tradition* (0.39). Another example is *Power*, that strongly correlates with *Achievement* (0.77), followed by *Security* (0.53), and weakly correlates with *Universalism* (0.36), *Benevolence* (0.37), and *Hedonism* (0.38) (Table 5).

**Table 5:** Ten basic human values correlations with civic participation.

	Civic participation	Tradition	Conformity	Benevolence	Universalism	Security	Self-direction	Power	Achievement	Hedonism	Stimulation
*Civic Participation*	1.00										
*Tradition*	−0.35	1.00									
*Conformity*	−0.25	0.63	1.00								
*Benevolence*	0.06	0.66	0.51	1.00							
*Universalism*	0.14	0.59	0.49	0.84	1.00						
*Security*	−0.38	0.76	0.62	0.61	0.55	1.00					
*Self-direction*	0.23	0.39	0.30	0.69	0.73	0.42	1.00				
*Power*	−0.28	0.45	0.43	0.37	0.36	0.53	0.45	1.00			
*Achievement*	−0.19	0.38	0.36	0.41	0.42	0.49	0.54	0.77	1.00		
*Hedonism*	0.06	0.36	0.15	0.55	0.54	0.36	0.58	0.38	0.48	1.00	
*Stimulation*	0.18	0.19	0.23	0.46	0.53	0.26	0.67	0.49	0.60	0.57	1.00

All correlations are significant at *p*-value ≤ 0.05.

Finally, the values also correlate directly with the indicators of wellbeing, such that *Tradition*, *Conformity* and *Security* show a negative association with *health*, while *Universalism*, *Self-direction*, *Achievement*, *Hedonism* and *Stimulation* show a positive one. As for *happiness* and *life satisfaction*, both are positively correlated with *Benevolence* and *Self-direction*, but negatively correlated with *Tradition*, *Security*, *Power*, *Achievement* and *Hedonism*. Additionally, *life satisfaction* positively correlates with *Universalism* (Table 6).

**Table 6:** Ten basic human values correlations with wellbeing.

	Health	Happiness	Life satisfaction
*Tradition*	−0.29	−0.09	−0.12
*Conformity*	−0.18	ns	ns
*Benevolence*	ns	0.08	0.06
*Universalism*	0.07	ns	0.04
*Security*	−0.29	−0.12	−0.18
*Self-direction*	0.21	0.05	0.08
*Power*	ns	−0.20	−0.21
*Achievement*	0.04	−0.17	−0.22
*Hedonism*	0.09	−0.04	−0.06
*Stimulation*	0.32	ns	ns

All correlations are significant at *p*-value ≤0.05, except those marked with “ns” as non-significant.

### 4.2 Regression analysis

The estimations carried out using both the Random Effects Model and the Fixed Effects Model show that there is a bidirectional relationship between values and *civic participation*, so that both variables have a certain impact on one another. However, the impact that participation has on human values is considerably greater than the impact that values have on participation, in all cases. Although the coefficients of the Random Effects Model are probably magnified as it is not the model suggested by the Hausman test, we get more significant results so that it is possible to observe the direction of the relationship between variables more clearly. Furthermore, we have repeated all the estimates using the Fixed Effects Model suggested by the Hausman test and, with slightly more conservative coefficients, the results are confirmed.

Additionally, when grouping the values in their 4 higher-order dimensions, the same direction of the relationship is observed for *Conservation* and *Self-enhancement*, the impact being negative in both cases. Also, *civic participation* has a positive effect on *Openness to Change*.

Similar to what is observed in the correlations (Table 5), the values on which *civic participation* has a positive impact are *Benevolence*, *Universalism*, *Self-direction*, *Stimulation*, and *Hedonism*, while its effect is negative on *Achievement*, *Power*, *Security*, *Conformity*, and *Tradition*. Additionally, *civic participation* seems to have a greater impact on *Power* (−) than on the remaining values (Table 7).

**Table 7:** Cross-lagged model for civic participation and the 10 basic human values.

Civic participation	Random effects		Fixed effects	
	Coef.	*p*-value	Coef.	*p*-value
CivicParticipation _t−1_ – CivicParticipation_t_	0.71	0.00	ns	ns
Benevolence_t_ – CivicParticipation_t_	0.02	0.00	0.02	0.00
CivicParticipation_t_ – Benevolence_t_	0.15	0.00	0.18	0.00
Benevolencet _t−1_ – CivicParticipation_t_	ns	ns	ns	ns
CivicParticipation _t−1_ – Benevolence_t_	ns	ns	ns	ns
Universalism_t_ – CivicParticipation_t_	0.03	0.00	0.02	0.00
CivicParticipation_t_ – Universalism_t_	0.26	0.00	0.22	0.00
Universalism _t−1_ – CivicParticipation_t_	ns	ns	ns	ns
CivicParticipation _t−1_ – Universalism_t_	0.18	0.00	ns	ns
Self-direction_t_ – CivicParticipation_t_	0.03	0.00	0.02	0.00
CivicParticipation_t_ – Self-direction_t_	0.35	0.00	0.20	0.00
Self-direction _t−1_ – CivicParticipation_t_	0.02	0.00	ns	ns
CivicParticipation _t−1_ – Self-direction_t_	0.24	0.00	ns	ns
Stimulation_t_ – CivicParticipation_t_	0.01	0.02	ns	ns
CivicParticipation_t_ – Stimulation_t_	0.20	0.00	ns	ns
Stimulation _t−1_ – CivicParticipation_t_	ns	ns	ns	ns
CivicParticipation _t−1_ – Stimulation_t_	0.12	0.00.	0.13	0.01
Hedonism_t_ – CivicParticipation_t_	0.02	0.00	0.02	0.00
CivicParticipation_t_ – Hedonism_t_	0.17	0.00	0.18	0.00
Hedonism _t−1_ – CivicParticipation_t_	ns	ns	ns	ns
CivicParticipation _t−1_ – Hedonism_t_	ns	ns	ns	ns
Achievement_t_ – CivicParticipation_t_	−0.02	0.00	−0.02	0.00
CivicParticipation_t_ – Achievement_t_	−0.23	0.00	−0.14	0.00
Achievement _t−1_ – CivicParticipation_t_	−0.03	0.00	−0.02	0.00
CivicParticipation _t−1_ – Achievement_t_	−0.29	0.00	−0.20	0.00
Power_t_ – CivicParticipation_t_	−0.05	0.00	−0.04	0.00
CivicParticipation_t_ – Power_t_	−0.41	0.00	−0.31	0.00
Power _t−1_ – CivicParticipation_t_	−0.03	0.00	−0.02	0.00
CivicParticipation _t−1_ – Power_t_	−0.33	0.00	−0.18	0.00
Security_t_ – CivicParticipation_t_	−0.03	0.00	ns	ns
CivicParticipation_t_ – Security_t_	−0.50	0.00	ns	ns
Security _t−1_ – CivicParticipation_t_	−0.03	0.00	ns	ns
CivicParticipation _t−1_ – Security_t_	−0.54	0.00	ns	ns
Conformity_t_ – CivicParticipation_t_	−0.03	0.00	−0.02	0.00
CivicParticipation_t_ – Conformity_t_	−0.34	0.00	−0.16	0.00
Conformity _t−1_ – CivicParticipation_t_	−0.02	0.00	ns	ns
CivicParticipation _t−1_ – Conformity_t_	−0.29	0.00	ns	ns
Tradition_t_ – CivicParticipation_t_	−0.03	0.00	ns	ns
CivicParticipation_t_ – Tradition_t_	−0.43	0.00	ns	ns
Tradition _t−1_ – CivicParticipation_t_	−0.02	0.00	ns	ns
CivicParticipation _t−1_ – Tradition_t_	−0.42	0.00	ns	ns

Regressions have been performed for all value-civic participation associations (also Dimension-civic participation), including present on present, past on past, and past on present. These have been omitted for space purposes but are available upon request. “ns” means non-significant.

This section also presents the results of the estimations with both models (Random and Fixed Effects) for each value per wellbeing indicator. In this case, although the coefficients of the Fixed Effects Model are considerably more conservative than those of the Random Effects Model, the directional pattern is also confirmed (Table 8).

#### 4.2.1 Health

The relationship between *health* and all values is bidirectional, but the impact that health has on human values is stronger than the impact that values have on health. Also, the values on which *health* has a positive effect are *Self-direction*, *Stimulation*, and *Hedonism*, while the relationship is negative for *Benevolence*, *Universalism*, *Security*, *Conformity*, and *Tradition*. And, the values on which *health* has the greatest impact are *Tradition* (−), *Security* (−), *Benevolence* (−) and *Stimulation* (+).

#### 4.2.2 Happiness

Again, the direction of the relationship is confirmed for all values, with *happiness* having a greater impact on human values than vice versa. For this case, *happiness* shows a positive effect on *Benevolence*, *Universalism* and *Self-direction* and a negative effect on *Stimulation*, *Achievement* and *Power*. In addition, the values on which *happiness* has the greatest impact are *Power* (−), and *Benevolence* (+).

#### 4.2.3 Life satisfaction

Similar to the other indicators of wellbeing, *life satisfaction* also has a stronger impact on human values than values have on satisfaction. *Life satisfaction* has a positive effect on *Benevolence*, *Universalism* and *Self-direction*, and an overall negative effect on *Power* and *Achievement*. Additionally, *life satisfaction* has a greater impact on *Benevolence* (+) than on the other values (Table 8).

**Table 8:** Cross-lagged model for health, happiness and life satisfaction, and the 10 basic human values.

Wellbeing	Health				Happiness				Life Satisfaction			
	Random effects		Fixed effects		Random effects		Fixed effects		Random effects		Fixed effects	
	Coef.	*p*-value	Coef.	*p*-value	Coef.	*p*-value	Coef.	*p*-value	Coef.	*p*-value	Coef.	*p*-value
Wellbeing_t−1_ – Wellbeing_t_	0.63	0.00	−0.08	0.00	0.39	0.00	−0.05	0.01	0.52	0.00	−0.01	0.53
Benevolence_t_ – Wellbeing_t_	−0.02	0.00	−0.03	0.00	0.02	0.00	0.02	0.00	0.03	0.00	0.03	0.00
Wellbeing_t_ – Benevolence_t_	−0.14	0.00	−0.30	0.00	0.30	0.00	0.31	0.00	0.27	0.00	0.33	0.00
Benevolence _t−1_ – Wellbeing_t_	−0.02	0.00	−0.02	0.00	ns	ns	ns	ns	ns	ns	ns	ns
Wellbeing _t−1_ – Benevolence_t_	ns	ns	−0.21	0.00	ns	ns	ns	ns	ns	ns	ns	ns
Universalism_t_ – Wellbeing_t_	ns	ns	−0.01	0.03	0.01	0.04	0.01	0.04	0.02	0.00	0.02	0.00
Wellbeing_t_ – Universalism_t_	ns	ns	−0.12	0.03	0.11	0.04	0.12	0.04	0.17	0.00	0.21	0.00
Universalism _t−1_ – Wellbeing_t_	ns	ns	−0.01	0.01	ns	ns	ns	ns	ns	ns	ns	ns
Wellbeing _t−1_ – Universalism_t_	ns	ns	−0.23	0.00	ns	ns	ns	ns	ns	ns	ns	ns
Self-direction_t_ – Wellbeing_t_	0.01	0.04	ns	ns	0.01	0.02	0.01	0.04	0.02	0.00	0.02	0.00
Wellbeing_t_ – Self-direction_t_	0.25	0.00	ns	ns	0.13	0.02	0.12	0.04	0.23	0.00	0.23	0.00
Self-direction _t−1_ – Wellbeing_t_	ns	ns	−0.01	0.01	ns	ns	ns	ns	ns	ns	ns	ns
Wellbeing _t−1_ – Self-direction_t_	0.18	0.00	−0.18	0.00	ns	ns	ns	ns	ns	ns	ns	ns
Stimulation_t_ – Wellbeing_t_	0.05	0.00	0.03	0.00	ns	ns	ns	ns	0.02	0.00	0.02	0.00
Wellbeing_t_ – Stimulation_t_	0.50	0.00	0.27	0.00	ns	ns	ns	ns	0.13	0.01	0.19	0.00
Stimulation _t−1_ – Wellbeing_t_	0.01	0.03	ns	ns	−0.02	0.00	−0.02	0.00	−0.01	0.04	ns	ns
Wellbeing _t−1_ – Stimulation_t_	0.31	0.00	ns	ns	−0.15	0.01	−0.17	0.00	−0.21	0.00	−0.21	0.00
Hedonism_t_ – Wellbeing_t_	0.01	0.04	ns	ns	0.01	0.00	0.03	0.00	0.02	0.00	0.03	0.00
Wellbeing_t_ – Hedonism_t_	0.12	0.02	ns	ns	0.23	0.00	0.32	0.00	0.24	0.00	0.37	0.00
Hedonism _t−1_ – Wellbeing_t_	ns	ns	ns	ns	ns	ns	ns	ns	ns	ns	ns	ns
Wellbeing _t−1_ – Hedonism_t_	ns	ns	−0.18	0.00	−0.19	0.00	−0.14	0.03	−0.18	0.00	ns	ns
Achievement_t_ – Wellbeing_t_	ns	ns	ns	ns	−0.02	0.00	ns	ns	−0.02	0.00	ns	ns
Wellbeing_t_ – Achievement_t_	ns	ns	ns	ns	−0.19	0.00	ns	ns	−0.22	0.00	ns	ns
Achievement _t−1_ – Wellbeing_t_	ns	ns	ns	ns	−0.03	0.00	−0.02	0.00	−0.03	0.00	−0.02	0.00
Wellbeing _t−1_ – Achievement_t_	ns	ns	ns	ns	−0.35	0.00	−0.25	0.00	−0.45	0.00	−0.30	0.00
Power_t_ – Wellbeing_t_	0.02	0.01	0.02	0.00	−0.04	0.00	−0.03	0.00	−0.03	0.00	−0.02	0.01
Wellbeing_t_ – Power_t_	0.09	0.03	0.14	0.00	−0.37	0.00	−0.29	0.00	−0.27	0.00	−0.14	0.01
Power _t−1_ – Wellbeing_t_	ns	ns	ns	ns	−0.04	0.00	−0.03	0.00	−0.04	0.00	−0.02	0.00
Wellbeing _t−1_ – Power_t_	−0.08	0.05	ns	ns	−0.33	0.00	−0.23	0.00	−0.40	0.00	0.27	0.00
Security_t_ – Wellbeing_t_	−0.03	0.00	−0.02	0.00	ns	ns	0.01	0.01	ns	ns	0.01	0.00
Wellbeing_t_ – Security_t_	−0.47	0.00	−0.26	0.00	ns	ns	0.17	0.01	ns	ns	0.20	0.00
Security _t−1_ – Wellbeing_t_	−0.02	0.00	ns	ns	−0.01	0.00	ns	ns	−0.01	0.00	ns	ns
Wellbeing _t−1_ – Security_t_	−0.50	0.00	−0.21	0.00	−0.27	0.00	ns	ns	−0.36	0.00	−0.14	0.05
Conformity_t_ – Wellbeing_t_	−0.02	0.00	ns	ns	ns	ns	ns	ns	0.01	0.01	0.02	0.00
Wellbeing_t_ – Conformity_t_	−0.23	0.00	ns	ns	ns	ns	ns	ns	0.13	0.02	0.19	0.00
Conformity _t−1_ – Wellbeing_t_	−0.02	0.00	ns	ns	ns	ns	ns	ns	ns	ns	ns	ns
Wellbeing _t−1_ – Conformity_t_	−0.35	0.00	−0.23	0.00	ns	ns	ns	ns	−0.11	0.05	ns	ns
Tradition_t_ – Wellbeing_t_	−0.04	0.00	−0.03	0.00	ns	ns	0.02	0.00	ns	ns	0.02	0.00
Wellbeing_t_ – Tradition_t_	−0.45	0.00	−0.26	0.00	ns	ns	0.18	0.00	ns	ns	0.20	0.00
Tradition _t−1_ – Wellbeing_t_	−0.04	0.00	−0.02	0.00	ns	ns	0.01	0.01	ns	ns	0.01	0.03
Wellbeing _t−1_ – Tradition_t_	−0.45	0.00	−0.23	0.00	−0.14	0.01	ns	ns	−0.18	0.00	ns	ns

Variable *Wellbeing* comprises 3 indicators: *Health*, *Happiness* and *Life Satisfaction*. Regressions have been performed for all value-wellbeing associations (also Dimension-wellbeing), including present on present, past on past, and past on present. These have been omitted for space purposes but are available upon request. “ns” means non- significant.

## 5 Discussion

The global trend of an aging population raises the question as to what strategies can help older people achieve the highest possible levels of wellbeing, as well as benefiting their communities from more participatory and democratic processes. Civic engagement has provided evidence of being part of the active and healthy aging paradigm and of promoting such wellbeing among older Europeans (Vega-Tinoco et al., 2021, 2022). Human values, as reflected in the theory proposed by Schwartz (1992), provide some insight into what motivates such participation, as well as their relationship with wellbeing.

From the analysis carried out we conclude that the direction of the relationship points to civic participation having more weight on values than values on participation. This means that, although certain values may motivate an older adult to participate civically, it is more likely that such participation has a more substantial influence on that person’s values. Thus, for example, the more importance a person assigns to Universalism, the more likely it is that they will engage in participatory activities, but if that person does participate, the more likely it is that their Universalism value will be positively reinforced. Conversely, the more importance an older person places on Power, the less likely they are to participate. However, if this person does in fact participate, it is very likely that the importance they assign to Power will be reduced.

Something similar happens with wellbeing: wellbeing has more impact on values than values have on wellbeing. While it is true that a person with a tendency towards certain values may experience a certain level of wellbeing, it is more likely that their level of wellbeing will have a stronger influence on their values. For example, an older person who believes Self-direction is important is likely to increase their level of happiness, but it is even more probable that a person who feels happy assigns greater importance to Self-direction. Likewise, the more importance an older adult places on Achievement, the less likely they are to enhance their happiness, but if the person already feels happy, then the importance they place on Achievement will probably decrease.

This last example could also be viewed from the perspective of a vicious cycle: the more importance an older person assigns to Achievement, the less happiness they will experience and, in turn, if that person’s happiness levels are low, the more importance they will place on Achievement. Thus, since the ultimate goal is wellbeing, civic engagement could act as an external agent to interrupt the cycle and alter both values and wellbeing for the better. Thus, if an older adult within the circle begins to participate, their drive towards Achievement could be reduced and in addition their happiness could increase directly due to such participation, thereby changing the direction of the cycle towards greater wellbeing.

In summary, through participation one could foster those values that will in turn increase wellbeing, especially given that civic participation has a positive impact on the Openness to Change dimension, which contributes the most to wellbeing, according to the results of this study as well as those found by Sortheix and Schwartz (2017) and Grosz et al. (2021). Therefore, civic participation should be promoted since it directly increases wellbeing, and moreover reinforces those values that positively influence the wellbeing of older people.

Also, the positive association of civic participation with Self-transcendence (Benevolence and Universalism), and negative association with Self-enhancement (Achievement and Power), are in agreement with the scientific literature (Pepper et al., 2009; Plagnol and Huppert, 2010; Schwartz, 2010; Bathini and Vohra, 2013; Hamby and Farrell, 2022). However, in addition, we find that civic engagement has a positive relationship with all values that are oriented towards Growth, and negative with those that are oriented towards Self-protection. This would also help to explain why civic participation has a positive effect on subjective wellbeing, as Growth-oriented values increase it (Grosz et al., 2021).

The study by Lee et al. (2021) suggests that the values-behavior relationship may be stronger than previously thought, depending on the importance assigned to the value. Thus, a person who assigns a high importance to a certain value will engage in behaviors that express that value. Moreover, the more importance a value acquires, the stronger this relationship becomes. Conversely, if a person attaches low importance to a value, they will engage less frequently in behaviors that express that value and, as its importance declines, the relationship weakens. Similarly, Ariza-Montes et al. (2017) assert that Human Resources professionals and non-profit organizations should ensure consistency between the values of their volunteers and the nature of the activities they perform, so as to increase their engagement and motivation.

Additionally, the results of the present study are in line with those of Hamby and Farrell (2022), who argue that an individual’s value orientation plays an important role in determining the wellbeing benefits that person receives from engaging in transformative service experiences, i.e., volunteer and community service activities. Thus, if a person participates in these types of activities because they value thrill-seeking, this person will most likely experience different changes in wellbeing than another person who participates because they seek self-fulfillment and personal growth.

It is however worth mentioning that our main results on the direction of the relationship between values and wellbeing do not entirely coincide with those of the panel data study by Grosz et al. (2021), where both directions of the relationship were also found to be significant, but neither seemed to be predominant over the other. However, the difference may lie in the fact that our sample comprises only older people, and also that these authors’ study only takes into account the Openness to Change dimension, and their sample is limited to Germany.

Furthermore, from the most recent theoretical perspective and based on ample empirical evidence (see Sortheix and Schwartz, 2017; Grosz et al., 2021) it is assumed that the relationship between wellbeing and values is given by the motivational orientation (Growth versus Self-protection) and the interest (personal versus social focus) of the value. Under this perspective, it is believed that values oriented to Growth increase subjective wellbeing, while those oriented to Self-protection reduce it. Also, values with a personal focus increase subjective wellbeing, but those with a social focus reduce it. Under this assumption, the Openness to Change dimension, which crosses both the Growth motivation and personal focus, increases wellbeing, as evidenced by Grosz et al. (2021). Conversely, Conservation values are assumed to reduce wellbeing, while Self-transcendence and Self-enhancement present a complex association by mixing both positive and negative influences of factors (Sortheix and Schwartz, 2017).

The results presented here mostly confirm this theory. However, the present study adds some further nuances to the interpretation. For example, the effect of motivational orientation is consistent, but only with the health indicator, so that values with social focus reduce subjective health, while those with personal focus increase it. However, this is not as accurate for happiness and life satisfaction, where Self-transcendence values increase both indicators even though they belong to the social focus. Thus, it seems important to clearly define what is considered as subjective wellbeing and it would also be desirable to include more than one wellbeing indicator in future research.

In addition, the results also highlight that, as people age, they tend towards greater Conservation and less Self-enhancement and Openness to Change. Consistently, Ariza-Montes et al. (2017), found that the prioritization of Self-enhancement and Openness to Change decreases across generational cohorts to reach its lowest levels in the older age group, while Conservation value importance increases, especially for Tradition, Conformity and Security. The authors indicate that Self-transcendence does not show significant differences throughout the lifespan, as in this study.

Along these lines, earlier research by Egri and Ralston (2004) found that Baby Boomers assigned greater importance to Openness to Change, while the Silent Generation did so for the opposite values, Conservation. Also, Baby Boomers attributed more importance to Self-enhancement than the Silent Generation, whereas, again, no meaningful generational differences were found in relation to Self-transcendence. These authors argue that the value orientation of a certain generational cohort is influenced by the major significant events that occurred in their pre-adulthood. However, they also note that Life Stage Theory suggests that values between generations follow a pattern related to the life cycle and that, as a person ages, they become more collectivist (social focus), conservative and self-transcendent, and less individualistic (personal focus), open to change or interested in self-enhancement.

Therefore, in view of the results presented above, the hypotheses would stand as follows:

*H1:* The relationship between civic participation and human values is bidirectional, with the impact of values on participation being stronger than vice versa. Partially accepted: the relationship is bidirectional but civic participation has a stronger impact on values, rather than the other way around.

*H2:* The relationship between wellbeing and human values is bidirectional, with the impact of values on wellbeing being stronger than vice versa. Partially accepted: the relationship is bidirectional but wellbeing has a stronger impact on values, rather than the other way around.

*H3:* Self-transcendence values promote civic participation, while Self-enhancement values hinder it. Accepted. Moreover, all Growth-oriented values (Self-transcendence and Openness to Change) promote civic participation, whereas Self-protection values (Self-enhancement and Conservation) hinder it.

*H4:* Openness to Change values promote wellbeing, Conservation hinders it, and Self-transcendence and Self-enhancement present a mixed association (both positive and negative). Accepted.

## 6 Limitations and further research

Among the limitations of the present study we find that the data used are cross-sectional. This issue is mitigated through the creation of the pseudo-panel, although it would be optimal to contrast results with authentic panel data in order to confirm inferences. Any future research that may provide additional information on the values-civic participation and the values-wellbeing directional association will surely allow for better interpretations.

In addition, the limitations inherent to the pseudo-panel methodology itself are to be considered. The main constraint is the variability of responses within each cohort, especially when the number of cohort members is small. However, fortunately most cohorts in this study contain a large number of members and, moreover, according to Deaton (1985), the errors-in-variables are likely to be more apparent than real. Additionally, by using both Random and Fixed Effects Models, we can confirm that the interpretation of the results is along the same lines.

Another limitation is the lack of data for all waves in many European countries, and even more so when by creating the pseudo-panel the number of observations is considerably reduced. For example, from the Mediterranean group we could only include Portugal and Spain. However, once the direction of the relationships is evidenced, the cross-sectional data can be used to make comparisons between European countries as future research, or at least between European welfare systems.

Furthermore, this study has only considered persons born before 1965 as it is focused on older people. However, it would be desirable to repeat it for all age groups and determine whether the results are maintained or vary according to age. In addition, we also propose to replicate this study by including the tenth wave of the European Social Survey (2021–2022), once it is available to the public, as it will show the effects that the COVID-19 pandemic has had on values, participation and wellbeing.

## Data availability statement

The original contributions presented in the study are included in the article/supplementary material, further inquiries can be directed to the corresponding author.

## Ethics statement

Ethical approval was not required for the study involving humans in accordance with the local legislation and institutional requirements. Written informed consent to participate in this study was not required from the participants or the participants’ legal guardians/next of kin in accordance with the national legislation and the institutional requirements.

## Author contributions

AV-T: Conceptualization, Data curation, Formal analysis, Methodology, Visualization, Writing – original draft. JS-G: Data curation, Formal analysis, Methodology, Visualization, Writing – review & editing. MG-L: Funding acquisition, Resources, Supervision, Validation, Writing – review & editing. MSB: Data curation, Visualization, Writing – review & editing. AG-L: Conceptualization, Methodology, Software, Supervision, Validation, Writing – review & editing.
